# Preservation and Tissue Handling Technique on Iatrogenic Dural Tear with Herniated Nerve Root at Cauda Equina Level

**DOI:** 10.1155/2016/4903143

**Published:** 2016-12-29

**Authors:** Ahmad Jabir Rahyussalim, Yoshi Pratama Djaja, Ifran Saleh, Ahmad Yanuar Safri, Tri Kurniawati

**Affiliations:** ^1^Department of Orthopaedic and Traumatology, Faculty of Medicine, Universitas Indonesia, Dr. Cipto Mangunkusumo General Hospital, Jakarta 10320, Indonesia; ^2^Neurophysiology Division, Neurology Department, Faculty of Medicine, Universitas Indonesia, Dr. Cipto Mangunkusumo General Hospital, Jakarta 10320, Indonesia; ^3^Stem Cell and Tissue Engineering Cluster, MERC Faculty of Medicine, University of Indonesia, Dr. Cipto Mangunkusumo Hospital, Jakarta, Indonesia

## Abstract

Iatrogenic or incidental dural tear is a relatively common complication in lumbar decompression surgery. Although mostly there are no changes that occurred in long-term result following an incidental durotomy, the sequelae are not always benign especially when the herniated nerve root is involved. Preservation and tissue handling is paramount in order to prevent further injury. Two cases of dural tear with herniated nerve root complicating the lumbar decompression surgery are presented. Direct watertight repair was performed using the preservation and tissue handling concept. Assessing the relative size between the dural tear and the root mass is the key in determining whether enlargement of tear is needed. Whenever feasible, the tear will not be enlarged. Opening the vent by using a suture anchor and manually repositioning the nerve root with a fine instrument is the key for an atraumatic handling of the herniated nerve root. Clinical and neurophysiology examination was performed postoperatively and no further neurologic deficit occurred despite the iatrogenic injury. Although some debate on a few intraoperative and postoperative details still persists, tissue handling and preservation concept should be applied in all cases.

## 1. Introduction

Iatrogenic or incidental dural tear is a relatively common complication in lumbar decompression surgery. The incidence varies and ranges from 1% to 17% [1–4]. The increased incidence of incidental durotomy (ID) is related to epidural fibrosis, which is induced by previous operation and advanced spinal degenerative changes, such as ossified yellow ligament [1, 3, 4]. Beside direct dural laceration, other intraoperative mechanisms causing dural tear are excessive nerve root traction during removal of the disc extrusion and excessive force during removal of the adherent yellow ligament [2].

Some authors reported that there are no changes that occurred in long-term results following a spine surgery complicated by an incidental durotomy [1, 4, 5]. However, the sequelae after a dural tear are not always benign. In several cases, the direct complications of dural tear include postural headache, meningeal pseudocyst, arachnoiditis, or meningeal infection. Indirect complications associated with prolonged bed rest frequently required in these cases are pneumonia, pressure ulcer, deep venous thrombosis, and pulmonary embolism.

Therefore, in order to reduce the risk of these complications, direct dural repair is recommended. The objectives of dural repair are containment of the nerve roots and creation of watertight closure to allow early mobilization of the patients, even though a period of bed rest is usually recommended after the repair of durotomy [6].

Various dural repair techniques have been described, such as direct primary suture using either simple interrupted suture or running locked suture, sealants such as fibrin glue, hydrogel or cyanoacrylate, bioabsorbable staples, and various types of grafts and patches [6]. Among these techniques, direct primary suture using simple interrupted suture is considered as the gold standard for achieving microsurgical anastomosis.

Despite the achievement of a watertight dural closure, several cases with herniated nerve roots are commonly associated with poor outcomes [7]. Accordingly, atraumatic tissue handling is paramount in order to avoid further injury to the nerve [8].

## 2. Case Illustration

### 2.1. Case  1

Female, 63-year-old, came with chronic back and leg pain, especially on her right side, due to spondylolisthesis at L4-5 and lumbar degenerative disc disease. On initial presentation, minor sensory deficit was found at the dermatomes of L4 and L5. Decreased motor power also occurred in ankle dorsiflexion (L4) and long toe extensor (L5). The diagnosis was confirmed by radiological examination and MRI images that showed degenerative changes at lumbar region, including scoliosis de novo along with degenerative spondylolisthesis at L4-5 compressing the spinal cord, that is, the L4 and L5 nerve roots, especially on the right side. After a series of failed conservative treatments consisting of oral medication and physiotherapy, the patient finally underwent a lumbar decompression surgery.

Posterior stabilization using pedicle screw and rod system were performed to restore the lumbar coronal and sagittal balance prior to performing L4-5 laminectomy. During the laminectomy and flavectomy, iatrogenic durotomy occurred as an adherence of yellow ligament to the spinal cord. The lesion occurred at the left dorsolateral region of L4 with herniated nerve root.

To repose the herniated nerve root, one anchor suture of 6-0 polypropylene monofilament (Premilene; B Braun, Melsungen, Germany) was placed on the lateral edge of the vent. The anchor suture was pulled carefully to the lateral side in order to open the vent without increasing the size. The herniated nerve root was then manually reposed gently into the vent by using the tip of nontoothed fine forceps. Unfortunately, the extent of the herniated nerve root was greater than the size of the vent. Therefore, additional incision on the dura was required to allow gentle attempt to repose the herniated nerve root.

Simple interrupted suture of 6-0 polypropylene was performed to create a watertight dural closure. Subfascial drainage was placed and controlled in order to prevent excessive drainage.

The patient was recommended to remain in postoperative bed rest for 48 hours. There were no complaints associated with the sequelae of the dural tear, such as dizziness or headache. Additional neurologic deficit did not occur. On the contrary, sensory improvement occurred at the dermatomes of L4 and L5.

Three months after the surgery, nerve conduction velocity study yielded an improvement in the right peroneal nerve motor but there was some decrease in the right sural sensory nerve compared to the preoperative evaluation (Figure 3). EMG evaluation cannot be performed due to preoperative massive radiculopathy.

### 2.2. Case  2

Female, 60-year-old, presented with back pain that radiated to both of her lower extremities for about one year. From physical examination, no motoric deficit was found and only a minor sensory deficit over the dermatomes of L4-5 and L5-S1 was found. The diagnosis was confirmed by MRI images showing canal stenosis of L4-5 and L5-S1 due to degenerative disc disease.

Similar to the first case, conservative therapy was first applied for a minimum of 2 months. After 3 months, there was no significant improvement that it was decided to perform posterior stabilization along with minimal decompression surgery by partial laminectomy.

**Figure 1:**
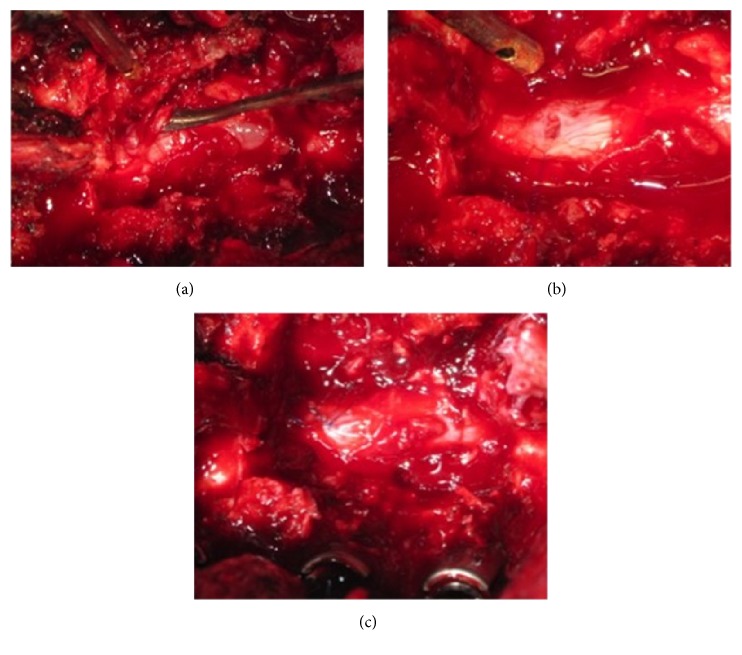
(a) Dorsal incidental durotomy with herniated nerve root (marked by root dissector). (b) Anchor suture to open the vent and reposing the herniated nerve root. (c) Watertight closure using simple interrupted 6-0 polypropylene.

Intraoperatively, during performing partial laminectomy, an iatrogenic durotomy occurred when removing the lamina and yellow ligament using a kerrison rongeur. To clearly expose the site and tear area, partial laminectomy was converted to total laminectomy. It was discovered later that the tear was at the dorsal region accompanied with herniated nerve root (Figure 1(a)).

Similar technique with the first case was performed by using an anchor suture to open the vent and insert the herniated nerve root by using a root dissector in order to minimize further injury to the nerve root. Watertight closure procedure was performed by using a simple interrupted suture of 6-0 polypropylene.

The protocol was continued with bed rest for the first 48 hours after the surgery. On the followup, there was no headache or postural dizziness, the wound healed without any complications, and the sensory deficit found before the operation was improved.

## 3. Preservation and Tissue Handling Technique

**Figure 2:**
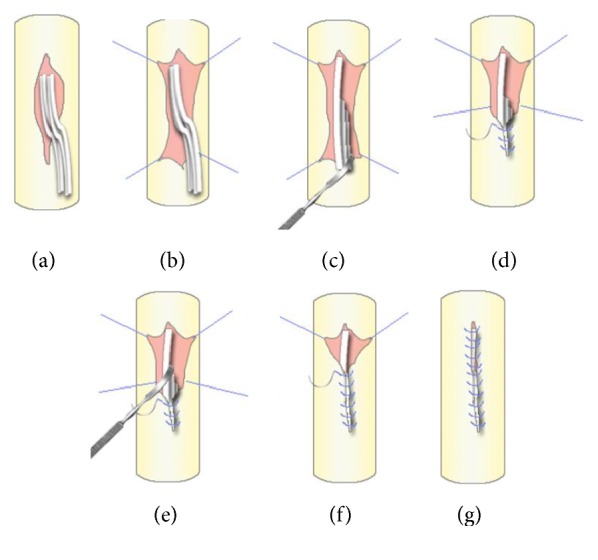
Tissue handling and preservation technique. (a) Assessment of the herniated nerve root; (b) putting the suture anchor at the edge of the vent; (c) reposing the nerve root at the top of the vent using blunt instrument; (d) gentle traction at the anchor to swallow the herniated nerve root and continuous suture were performed; (e) using the blunt edge of the root dissector to give a gentle push for the remaining herniated nerve root; (f) continuous suture was performed to the cephalad part of the vent; (g) final result shows that all nerves were into dural sac and the tear was recovered.

**Figure 3:**
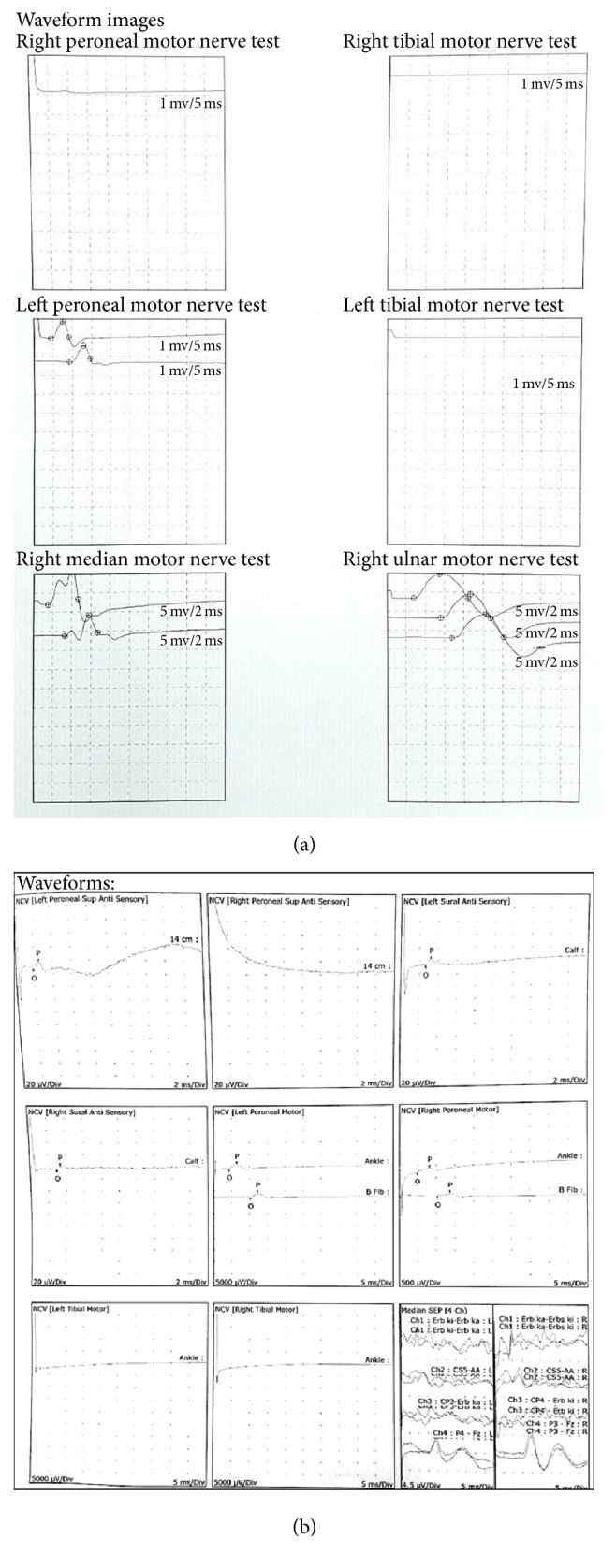
(a) Preoperative waveform images of nerve conduction velocity test in the first patient; (b) postoperative (3 months) waveform images showing improvements in the right peroneal nerve motor test and decrease in amplitude in the right sural nerve sensory test.

In order to achieve optimal care in dural repair, preservation and tissue handling are paramount. There are five general steps that should be taken into consideration (Figure 2). The first one is assessing the size of the tear and the ratio to the size of the herniated nerve root in order to determine whether or not the tear size should be enlarged. Second, preparation of microsurgery tools before the operation is essential in all lumbar decompression surgeries. Sealants are also needed in cases where tears are not repairable or watertight closures are not achieved.

Third, ensuring a sterile environment by gently cleansing the tear site before the repair should be done. Blood clot around the tear site may interfere with the dural repair process. Fourth, a watertight dural repair is performed whenever possible and the use of sealant is recommended as an adjunct. Fifth, the final step is ensuring the quality of the repair either by using valsalva maneuver or by neurophysiology assessment.

## 4. Discussion

As mentioned above, the incidence of incidental durotomy (ID) varies from 1% to 17%. However, herniation and incarceration of nerve roots due to the tear are quite rare, especially on nontrauma cases [9]. Herniated nerve roots in iatrogenic durotomy cases are commonly associated with poor prognosis [7]. Chang et al. summarize 7 reported cases of herniated nerve roots following a discectomy. Five out of the seven cases had their neurological deficit completely recovered and the remaining two were left with permanent neurologic deficit. Both cases had dural defects at the ventral side, in which surgical repair was quite challenging [9]. Fortunately, both cases of the present study had the tears at the dorsal and dorsolateral region of the cord, which were easier to repair and had more favorable outcomes compared to the above-mentioned series.

Gentle manipulation to the herniated nerve roots is mandatory to prevent further neurologic injury. The use of nontoothed fine forceps is commonly acknowledged to repose nerve roots. However, the use of a more concave side of the nerve root dissector is believed to be less traumatic in guiding the nerve roots back inside the vent. Further study is required to justify this concept.

Limited size of vent is also a problem when reposing the nerve roots. The dorsal dural vent was primarily repaired, surgically, by increasing the vent, and manually, by reposing the nerve roots. However, enlarging the vent is controversial and should be used as a last resort. Tewari and Gupta proposed another method to repose these nerve roots by using the “no touch hip flexion technique.” Without touching the nerve root or enlarging the vent, the herniated nerve roots are reposed indirectly by flexing the hip joint. By flexing the hip, intrathecal stretching of the nerves caused the herniated nerve roots to go inside spontaneously [10]. This technique will minimize further damage to the roots; however, it requires changing the position of the patient to semiprone on Jackson table or Wilson frame during the surgery, which could be quite troublesome for the surgical team members who are not familiar with this technique. Another alternative is by using a suture anchor on the edge of the vent as mentioned in the present series. The concept of this technique is maximizing the opening of the vent without increasing the size, so the herniated nerve root can be reposed manually and gently. However, in certain cases with the size of the herniated nerve roots being too large, in order to allow gentle and minimal manipulation to the nerve roots, an additional incision of the dura is unavoidable.

Watertight closure of the dural tear is mandatory whenever possible, by using either simple interrupted suture or continuous locked suture technique. Similar outcomes between these two suture techniques were found [11]. Cain Jr et al. [12] showed that there was no significant difference in the leak pressure in a dural repair model. A 6-0 prolene suture is a recommended product in dural repair. Using a suture with the closest diameter in relation to the needle's diameter is important to minimize leakage from the needle hole [6].

If a tight suture cannot be performed or the location is not accessible (ventral dural tear), the use of sealant, such as collagen patch, fibrin glue, or hydrogel, is recommended [6, 13]. Sealants are effective in reducing leak, especially when applied in the presence of suture.

Drainage is controversial in dural tear cases. Some recommend the absence of drainage to prevent excessive draining of CSF. Others recommend controlled drainage to prevent meningoceles and extradural hematomas [14]. In a protocol proposed by Wolff et al., the use of controlled drainage is recommended only if the suture is watertight. In nonclosed breaches, it is not clear whether drainage will result in fewer revisions. Furthermore, the low complication rate makes the value of drainage questionable when compared to the risk of cerebral complication due to excessive drainage of CSF [13].

Postoperative 48-hour bed rest is recommended following an iatrogenic durotomy repair. Bed rest is thought to reduce hydrostatic pressure on the repaired dura. According to postoperative protocol proposed by Khan et al., a trial of a brief bed rest (48 hours) followed by early mobilization was an effective strategy and was successful in 98.2% of their series [15]. Previous study by Wang et al. also stated similar outcome. In their study, an average bed rest period of 2.9 days resulted in only 2.3% reoperation rate (2 out of 88 patients) [4].

Postoperative neurophysiology evaluation was performed to evaluate the extent of the nerve injury/recovery related to the surgery (and iatrogenic durotomy). Improvement that was found in right peroneal nerve (motor) resulted from the decompression surgery. Meanwhile, there was some decrease in sural nerve (S1, 2) conductivity, which is possibly related to the injury. The limitation of this examination is that evaluation on the nerve conduction that innervates the autonomic element in accordance with the patient's complaint cannot be performed. Another limitation of this study is the minimal number of cases. Further evaluation, such as MRI and serial clinical evaluation, should be performed in the future.

## 5. Conclusion

When encountering an incidental durotomy with herniated nerve root during lumbar decompression surgery, preservation of the existing structure should be the first priority. This may be done by using a preservation and tissue handling technique by first assessing the size of the vent and, whenever feasible, opening the vent by anchoring using suture and reposing the nerve root by using a fine instrument. This way, further neurological damage can be prevented. Intraoperative and postoperative protocols are equally important to optimize the outcome and prevent future complications.

## References

[1] Cammisa F. P., Girardi F. P., Sangani P. K., Parvataneni H. K., Cadag S., Sandhu H. S. (2000). Incidental durotomy in spine surgery. *Spine*.

[2] Kalevski S. K., Peev N. A., Haritonov D. G. (2010). Incidental Dural Tears in lumbar decompressive surgery: incidence, causes, treatment, results. *Asian Journal of Neurosurgery*.

[3] Epstein N. E. (2007). The frequency and etiology of intraoperative dural tears in 110 predominantly geriatric patients undergoing multilevel laminectomy with noninstrumented fusions. *Journal of Spinal Disorders and Techniques*.

[4] Wang J. C., Bohlman H. H., Riew K. D. (1998). Dural tears secondary to operations on the lumbar spine. Management and results after a two-year-minimum follow-up of eighty-eight patients. *Journal of Bone and Joint Surgery A*.

[5] Jones A. A. M., Stambough J. L., Balderston R. A., Rothman R. H., Booth R. E. (1989). Long-term results of lumbar spine surgery complicated by unintended incidental durotomy. *Spine*.

[6] Dafford E. E., Anderson P. A. (2015). Comparison of dural repair techniques. *Spine Journal*.

[7] Ahn Y., Lee H. Y., Lee S.-H., Lee J. H. (2011). Dural tears in percutaneous endoscopic lumbar discectomy. *European Spine Journal*.

[8] Tewari V. K., Gupta H. K. D. (2014). Reposing the herniated spinal nerves following accidental iatrogenic dural tear in spine surgery-the ‘no touch hip flexion technique’. *Journal of Neurosciences in Rural Practice*.

[9] Chang M.-Y., Chan J.-Y., Huang C.-T., Liu Y.-K., Huang J.-S. (2012). Cauda equina incarceration secondary to dural tears after lumbar microsurgical discectomy. *Formosan Journal of Surgery*.

[10] Tewari V. K., Gupta H. K. D. (2014). Reposing the herniated spinal nerves following accidental iatrogenic dural tear in spine surgery-The ‘no touch hip flexion technique’. *Journal of Neurosciences in Rural Practice*.

[11] Schlechter B., Guyuron B. (1994). A comparison of different suture techniques for microvascular anastomosis. *Annals of Plastic Surgery*.

[12] Cain J. E., Dryer R. F., Barton B. R. (1988). Evaluation of dural closure techniques. Suture methods, fibrin adhesive sealant, and cyanoacrylate polymer. *Spine*.

[13] Wolff S., Kheirredine W., Riouallon G. (2012). Surgical dural tears: prevalence and updated management protocol based on 1359 lumbar vertebra interventions. *Orthopaedics and Traumatology: Surgery and Research*.

[14] Tafazal S. I., Sell P. J. (2005). Incidental durotomy in lumbar spine surgery: incidence and management. *European Spine Journal*.

[15] Khan M. H., Rihn J., Steele G. (2006). Postoperative management protocol for incidental dural tears during degenerative lumbar spine surgery: a review of 3,183 consecutive degenerative lumbar cases. *Spine*.

